# Growth modeling of the European grayling (*Thymallus thymallus* L.) in a large alpine river based on age‐at‐length, mark‐recapture, and length‐frequency data

**DOI:** 10.1111/jfb.16056

**Published:** 2025-01-11

**Authors:** Jan Droll, Christoffer Nagel, Joachim Pander, Sophie Ebert, Juergen Geist

**Affiliations:** ^1^ Aquatic Systems Biology Unit, TUM School of Life Sciences Technical University of Munich Freising Germany

**Keywords:** fish tagging, freshwater fish conservation, grayling, growth, life history, population dynamics

## Abstract

Animal growth is a fundamental component of population dynamics, which is closely tied to mortality, fecundity, and maturation. As a result, estimating growth often serves as the basis of population assessments. In fish, analysing growth typically involves fitting a growth model to age‐at‐length data derived from counting growth rings in calcified structures. Additionally, fish growth can be estimated using length‐frequency data or data on changes in length derived from mark‐recapture events. In our study of the European grayling (*Thymallus thymallus* L.) in the alpine region of Germany, we utilized all three types of datasets to develop the initial growth model. For the age‐at‐length data from scales, we applied the traditional von Bertalanffy growth function using both a Bayesian and a frequentist approach. Furthermore, we adopted the mark‐recapture data along with the Fabens model for reparametrizing the von Bertalanffy growth model. The electronic length‐frequency analysis (ELEFAN) was employed to examine the length‐frequency data of the grayling, encompassing multiple sampling events from 2013 to 2022. Our findings indicated that the mark‐recapture data, in conjunction with the Fabens model, yielded the most plausible values for both statistical approaches. When the von Bertalanffy growth function was used, the frequentist approach generated unreasonably high values, whereas the Bayesian version produced meaningful results when appropriate priors were applied, suggesting potential issues with the age‐at‐length data related to ageing. The ELEFAN approach produced the smallest yet reasonable growth parameters, contradicting other studies on the European grayling. The lower values may be attributed to the lack of larger fish in most of the sampling events, resulting in a relatively low asymptotic length and slow growth rate. As demonstrated in this case study on grayling from the River Inn, the use of growth characteristics may be a currently underestimated yet very useful indicator of target species assessment that can nicely complement other population health indicators.

## 1 INTRODUCTION

Growth is one fundamental factor in a fish's life as many biological processes governing population dynamics, like maturation, fecundity, egg quality, susceptibility to predation, and natural mortality, are related to size (Barneche et al., 2018; Lorenzen, 2016; Lorenzen & Enberg, 2002; Munch & Conover, 2003; Quist & Isermann, 2017; Saborido‐Rey & Kjesbu, 2012). Understanding these processes is crucial for conserving and managing fish populations, especially in freshwater, where populations are declining fast in response to multiple anthropogenic stressors, such as pollution, habitat fragmentation, and climate change (Dias et al., 2017; Matthaei & Lange, 2016). Therefore, estimating growth is usually one of the first steps in providing essential baseline data for assessing the status of fish populations as well as for their conservation and fisheries management.

In commercial fisheries, the growth of individual fish is usually estimated by age‐at‐length data, identified by removing otoliths and counting annuli, which form in most calcified structures in response to reduced growth due to lower temperatures in winter (Campana & Thorrold, 2001). Like many poikilotherms, fish show indefinite growth and annuli form throughout their lifetime (Popper & Lu, 2000). Otoliths used for aging are sampled lethally, which can conflict with conservation and management goals when the target species is threatened. Scales and fin rays are often considered a non‐lethal alternative (Quist & Isermann, 2017). Both structures can be sampled with minimal effort, regrow when removed, and have been intensively used for age determination (Koch & Quist, 2007; Maceina & Sammons, 2006). The accuracy of scales, however, is often the subject of fierce discussions because evaluation studies with scales from known‐age fish are rare and do not exist for many species (Quist & Isermann, 2017). In addition, sampling of fin rays and scales can be part of animal ethics and welfare considerations, often hampering an easy application. Typically, the obtained length‐at‐age data are fitted to a von Bertalanffy growth model (vBGM; [Von Bertalanffy, 1938]), which is widely used in fisheries to describe growth trajectories (Haddon, 2011). Annuli, however, only form in temperate areas when temperatures get low enough during the winter to reduce the growth of fish, which is not the case in tropical regions. To avoid this problem, Pauly et al. (1980) developed a method that analyses the length‐frequency of catches over time. Growth is typically normally distributed, also within age cohorts, which makes it possible to track these cohorts and their growth through time (Fournier, 1983). Compared to the “classical” approach, where the age of every single fish is identified, the length‐frequency analysis does not give any information about the age and/or length of an individual or the variability in length within an age cohort.

Data obtained from observations of the change in body length through mark‐recapture over time can offer an alternative opportunity to the previously described methods. Although mark and recapture methods are often used in fisheries to estimate abundance, mortality, or recruitment, few studies use length information obtained by mark and recapture setups to model the growth of fish. However, several reparameterizations of the vBGM are available to estimate the growth of fish based on those data (Fabens, 1965; Francis, 1988; Wang et al., 1995). Among the most used is the reparameterization after Fabens (1965), who modified the vBGM to describe the change in length as a function of length at the marking event and the time until the recapture. This method assumes that the mean growth declines linearly with increasing length. In the past, the method after Fabens (1965) has been used to describe the growth of skipjack tuna *Katsuwonus pelamis* L. (Hallier & Gaertner, 2006), finetooth shark *Carcharhinus isodon* (Valenciennes 1839) (Carlson et al., 2003), shovelnose sturgeon *Scaphirhynchus platorynchus* (Rafinesque 1820) (Hamel et al., 2015), and muskellunge *Esox masquinongy* (Mitchill 1824) (Sheffer et al., 2022).

Mark‐recapture studies are not commonly conducted with European freshwater fish species, particularly those of little or no commercial interest.

Once a widespread fish, the populations of the European grayling (*Thymallus thymallus* L.) (hereafter grayling) have steeply declined and still show negative trends in Germany, leading to a critically endangered evaluation on the red list of Germany (Freyhof et al., 2023). The substantial decline, together with its specific habitat requirements, makes the grayling a target species for conservation and restoration efforts. The grayling needs cool, well‐oxygenated water with sandy and gravelly grounds; therefore, the degradation of rivers caused by habitat deterioration plays a crucial role in the decline of grayling (Hayes et al., 2021; Marsh et al., 2022; Northcote, 1995). It is also discussed if its behavior makes the grayling especially vulnerable to the predation of piscivorous birds (Uiblein et al., 2001), but rising water temperatures due to climate change might play a more essential role in the decline of grayling populations than the predation through birds (Wedekind & Küng, 2010). Due to its critically endangered status, multiple studies on genetics (Gum et al., 2009, 2003) and habitat requirements (Mueller et al., 2018) have been carried out in the alpine region of Germany. However, basic information on population dynamics like growth, recruitment, and mortality is still missing.

Considering these challenges, the core objective of this study was to characterize the growth of the grayling from one of its last remaining strongholds in central Europe, the River Inn in Germany. Specifically, we hypothesize that scales provide acceptable results for the age of grayling but that the accuracy of the aging process will decline with increasing age of the individual.

## 2 METHODS

### 2.1 Study area

**FIGURE 1:**
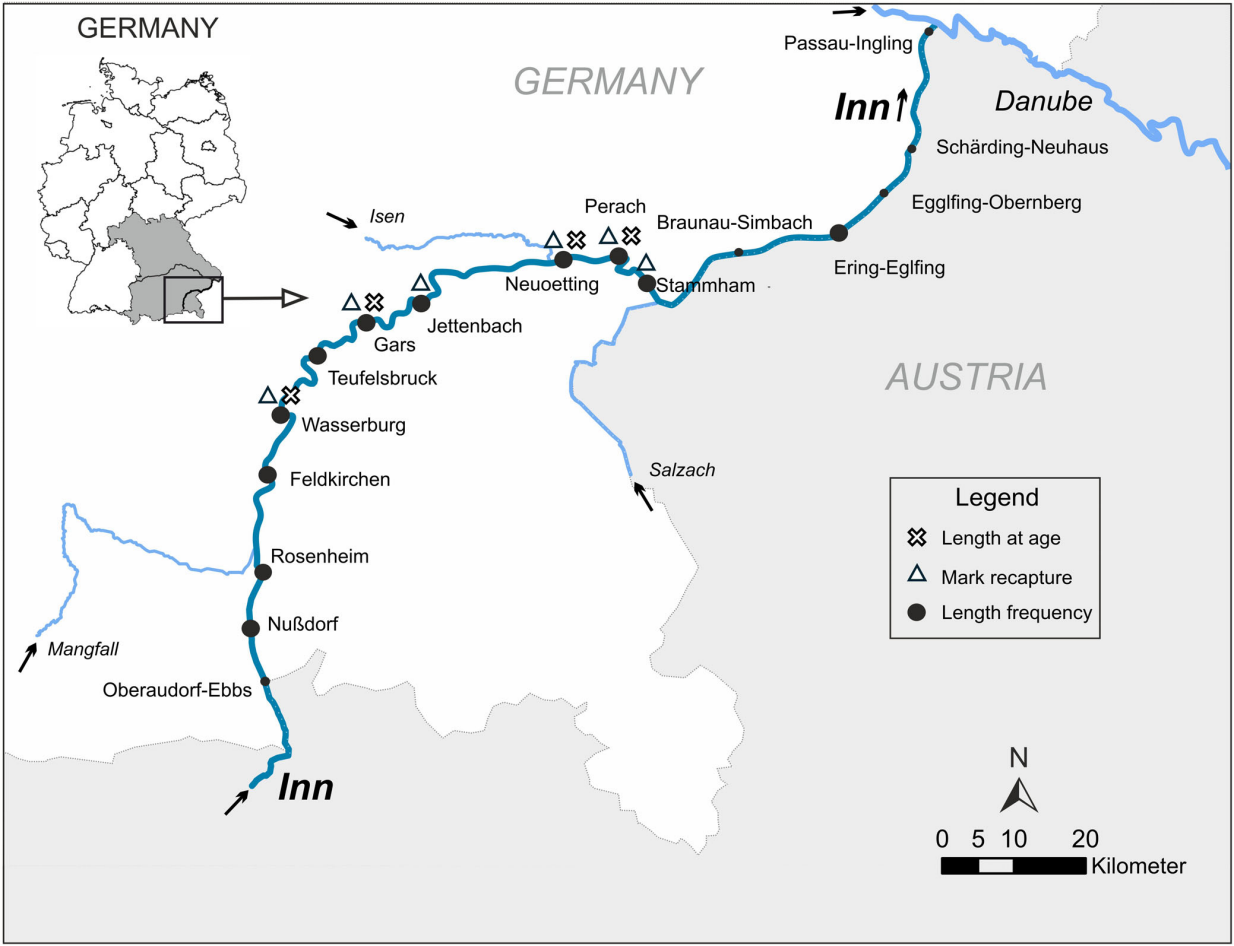
Map of the River Inn in the southeast of Germany and its main tributaries. The sampling sites for the different datasets are indicated with big black dots for the length‐frequency data; triangles indicate the sites where the mark and recapture data were recorded, and crosses indicate the sampling sites where scales of the grayling were collected. The small black points are placed at the position of a hydropower plant, with the associated name, respectively. Black arrows indicate the flow direction.

The study was performed in the River Inn (Figure 1), a large alpine river in the southeast of the state of Bavaria, which is the fourth‐largest river by discharge in Germany. The Inn has its source in the Engadin, Switzerland, and flows for over 517 km through Austria and Germany until it finally discharges into the Danube River at the city of Passau. Formally being a furcating river, bank stabilization and impoundment for flood protection and electricity production changed the river dramatically and restricted lateral and longitudinal connectivity. The River Inn is fed by meltwater of glaciers and snow due to its alpine catchment. This results in relatively low water temperatures throughout the year, a fluctuating discharge, and high loads of deferred sediments from spring to autumn.

The River Inn has been part of an extensive restoration campaign since 2013 to mitigate the effects of bank stabilization and impoundment. During this restoration project, life cycle–specific habitats for rheophilic fish have been restored, and river fragments were reconnected via fish passes. To assess the success of the restoration efforts, a large‐scale passive integrated transponder (PIT)‐tag project was initiated in 2020 to cover major parts of the German Inn (see Nagel et al., 2025, in review).

### 2.2 Data collection

We used three different datasets in this study: (1) age‐at‐length data, (2) mark‐recapture data, and (3) length‐frequency data. The age‐at‐length and the mark‐recapture data were collected between September 2022 and September 2023. Recurring transects of the River Inn between river km 173.1 and 75.4 were sampled regularly by electrofishing, including the mainstream and fish passes. Captured grayling were narcotized with tricaine methanesulfonate (MS‐222) and tagged with 12‐mm full duplex (FDX) PIT‐Tags (Biomark APT12, Boise, ID, USA). The total length to the closest millimetre was recorded using an electronic measuring board (Biomark Electronic Measuring Board Firmware v2.1.2 [100 cm] Boise). Before the tagging procedure, the grayling were checked for possible recaptures. Scales of 122 grayling obtained during the tagging process, in September 2022, were put into coin envelopes. The air‐dried scales were subsequently separated from each other and soaked in warm water for about 2 min. Any remaining mucus or skin was removed by either rubbing the scales between fingers or with tissue paper. Replacement scales identified by the absence or irregular patterns of circuli were not considered for further analysis. Afterwards, the dry but still flexible scales were placed between labeled glass slides. The prepared scales were viewed using a stereomicroscope (Olympus SZX10, Tokyo, Japan), and photographs of three of the prepared scales were taken using the software “cellSensEntry” (Olympus). Two readers without any knowledge about capture, date, location, or any biological information independently estimated the age of the fish for each of the three scales. Afterwards, the results of the aging process of both readers were used to calculate the mean age based on the three scales for further analysis. Grayling are known to spawn between March and April (Kottelat & Freyhof, 2007). To address the variability in age, we added an arbitrary hatching date (April 1) to the data and incorporated the additional day as a fraction of the year.

The third dataset consists of length‐frequency data collected during sampling for monitoring fish communities at the River Inn. The monitoring was performed by electrofishing standardized 30‐m hauls covering swaths of the German Inn and a wide variety of habitats (Pander & Geist, 2010). During the monitoring, the caught fish were identified, measured to the closest centimetre, and released afterwards. The data contain the length and frequency of grayling collected at 25 sampling events from autumn 2013 through spring 2022, usually sampled in spring, summer, and autumn. A winter sampling was included in the years 2019 and 2021. Due to the COVID pandemic, only the summer period could be sampled in 2020.

### 2.3 Data analysis

All analyses were performed in R version 4.0.4 (R Core Team, 2021). We used a frequentist and a Bayesian approach to model the growth of the grayling at the River Inn using non‐linear least squares (nls), as described in Ogle (2018) and Stan version 2.21.5 (Stan Development Team, 2022). To model the growth based on age‐at‐length data, we used the traditional von Bertalanffy growth model (vBGM) (Beverton & Holt, 2012; Quinn & Deriso, 1999):

(1) $$L_{t}=L_{\infty }\left[1-e^{-K\left(t-t_{0}\right)}\right]+\varepsilon_{i}$$

(2) $$\varepsilon_{i}\sim \text{normal}\left(0,\sigma \right)$$

where *L*_*t*_ is the length at time *t*, and $L_{\infty },K,$ and *t*_*0*_ are parameters that need to be estimated. *K* is the (Brody) growth coefficient and shows how fast the function approaches $L_{\infty }$. $L_{\infty }$ is the mean asymptotic length, whereas *t*_0_ is the length when the age is 0. In the case of the Bayesian model *ε*_*i*_ is a normally distributed random error term with a mean of 0 and a standard deviation of σ.

To estimate the growth based on the mark‐recapture data, we used a Bayesian and a frequentist version of the modified vBGM after Fabens (1965):

(3) $$L_{r}=L_{m}+\left(L_{\infty }-L_{m}\right)\left(1-e^{\left(-K{∆}_{t}\right)}\right)+\varepsilon_{i}$$

(4) $$\varepsilon_{i}\sim \text{normal}\left(0,\sigma \right)$$

where *L*_*r*_ is the length of the fish at the recapture event, *L*_*m*_ is the length of the fish when it was marked, and *Δ*_*t*_ is the time difference (year ^−1^) between the mark and the recapture events. $L_{\infty }$ and *K* are the parameters that need to be estimated and have a similar meaning as in the traditional vBGM. Also, with the Bayesian version of the growth model after Fabens *ε*_*i*_ represents a normally distributed random error like in the traditional vBGM. We used the Bayesian models with 50,000 iterations, of which 25,000 were discarded after the burn‐in phase, with a thinning interval of 10, and priors based on studies from other European regions (Table 1). The prior information on the different parameters of the growth models was used to construct normally distributed priors with mean values and SD based on the parameters of the studies shown in Table 1. 95% CIs for the frequentist models were estimated using the car package (Fox & Weisberg, 2019).

**TABLE 1:** Prior information used to fit Bayesian growth models to age‐at‐length and mark‐recapture data.

Reference	*L*_∞_	*K*	*t*_0_	Locality
Fish Base (Froese & Pauly, 2024)	375.00	0.214	−0.080	Kanin Peninsula (Russia)
Guillaud et al., 2017	544.00	0.190	−1.400	Historical data from Europe
Woolland and Jones (1975)	575.00	0.223	0.115	Llyn Tegid (1946–52) (Wales)
Woolland and Jones (1975)	456.00	0.386	0.222	Llyn Tegid (1962–68) (Wales)
Woolland and Jones (1975)	389.00	0.424	0.149	Upper Dee (Wales)
Woolland and Jones (1975)	417.00	0.484	0.281	Corwen (Wales)
Giri, 2017	437.80	0.238	0.540	Valåe (Norway)
Giri, 2017	438.80	0.238	0.488	Steinbekken (Norway)
Giri, 2017	393.33	0.292	0.557	Sandbekken (Norway)
Giri, 2017	418.12	0.241	0.490	Shyrjon (Norway)
Giri, 2017	456.41	0.220	0.520	Sørskottåe (Norway)

To estimate the growth based on the length‐frequency data, a non‐seasonal vBGM (see Equation 1) was fitted to the length‐frequency data with the ELEFAN (electronic length‐frequency analysis) approach (Pauly 1987). The model was fitted to the length‐frequency data using a genetic algorithm as optimization method (Mildenberger et al., 2017; Taylor & Mildenberger, 2017). Genetic algorithms are adaptive heuristic search algorithms that try to find the best fit based on principles of natural selection and genetics (Scrucca, 2013). The ELEFAN was fitted over the length‐frequency data via the genetic algorithm using the fishboot package (Schwamborn et al., 2019) (https://github.com/rschwamborn/fishboot, R package version 0.1), which offers additional 95% CIs to the estimated parameters via bootstrapping.

To enable a straightforward visual comparison, the parameters derived from the analysis were used to construct growth curves. To enhance comparability, the *t*_0_ values for both the von Bertalanffy growth function and the ELEFAN model were set to zero.

### 2.4 Software availability

The code used to analyse the data can be accessed on the first author's GitHub page (https://github.com/JanDroll/Grayling_growth). Additionally, we used the packages tidyverse (Wickham et al., 2019), ggridges (Wilke, 2021), patchwork (Pedersen, 2020), tidybayes (Kay, 2022), ggforce (Pedersen, 2022), and scico (Pedersen & Crameri, 2023) for the data analysis and visualization.

## 3 RESULTS

**FIGURE 2:**
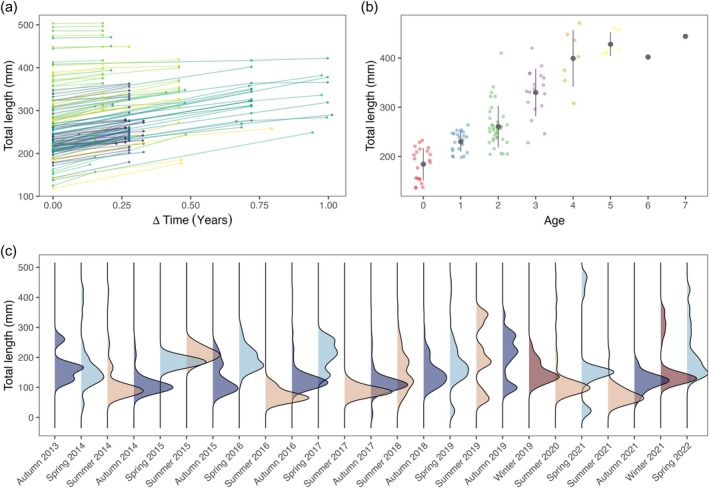
(a) Growth in length (mm) for capture‐mark‐recapture events over time; every line and colour stands for an individual fish, whereas a point indicates a capture event. (b) The individual and mean length, with SD, of fish per age was identified with scales, and the different colours indicate different age classes. (c) Kernel‐densities of the length distribution of grayling catches between autumn 2013 and spring 2022; the different seasons are marked by different colours (spring = light blue, summer = beige, autumn = dark blue, winter = red).

Overall, this study contained data from 2080 grayling; the scales of 122 grayling were used to estimate the length‐at‐age (dataset 1), 121 individuals were marked and recaptured after different time periods (dataset 2), and the length‐frequency data contained the length measurements of 1837 grayling (dataset 3). At the marking process, the smallest fish had a total length of 119 mm, whereas the largest individual was 503 mm. At the recapture events, the size of the individual fish ranged from 157 to 504 mm. The time between the mark and the recapture events ranged between 0.09 and 1.01 year^−1^ (35–370 days). Among the marked fish, smaller grayling grew faster than larger individuals (Figure 2a).

For the age‐at‐length data, the estimated age ranged from 0+ to 7+ years, whereas the length of the individuals ranged from 136 to 471 mm (Figure 2b). In general, the range of length per age cohort was relatively high, especially for age 2+, where the length had a range of 206 mm (204–410 mm), age 3+ with a range of 192 mm (228–420 mm), and for age 4+, where the range covered 163 mm (308–471 mm).

The length‐frequency data (dataset 3) comprehended the seasons spring, summer, and autumn (and in 2 years also winter) over 9 years (2013–2022). The length of the sampled fish ranged from 10 to 470 mm, whereas the largest fish were caught in the summer of 2020 and spring of 2021, and the smallest fish in the autumn of 2017 (Figure 2c).

### 3.1 Growth model results

The parameters estimated with the Bayesian and the frequentist approach of the growth model after Fabens were almost congruent for the asymptotic length and the growth coefficient (Figure 4). For *L*_∞_ the frequentist approach of the Fabens model estimated 538.85 mm, and the Bayesian approach estimated 536.82 mm. Values calculated for the growth coefficient *K* were 0.38 year^−1^ for the Bayesian Fabens model and 0.37 year^−1^ for the frequentist version of the model. The Bayesian version of the growth model showed a smaller credible interval (493.91–586.37 mm for *L*_∞_ and 0.25–0.49 year^−1^ for *K*) than the confidence interval of the frequentist Fabens model (474.32–678.30 mm for *L*_∞_ and 0.25–0.49 year^−1^ for *K*) (Table 2; Figure 4).

**TABLE 2:** Results of the estimated parameters by the three different growth models.

Method	*L*_∞_	*K*	Type
von Bertalanffy	524.46 (464.84–592.83)	0.21 (0.16–0.26)	Bayesian
von Bertalanffy	3620.30 (1914.94–10,078.97)	0.01 (0.005–0.03)	Frequentist
Fabens	536.82 (493.91–586.37)	0.38 (0.31–0.45)	Bayesian
Fabens	538.85 (474.32–678.30)	0.37 (0.25–0.49)	Frequentist
ELEFAN	436.50 (410.40–470.12)	0.12 (0.11–0.17)	Frequentist

*Note*: The von Bertalanffy model was fitted to age‐at‐length data, the growth model after Fabens was fitted to mark‐recapture data collected over a year, and the ELEFAN approach was used on length‐frequency data of 9 years. Uncertainty of the parameters is shown in the parentheses below the values. Bayesian models have credible intervals, and frequentist models have confidence intervals, respectively.

**FIGURE 3:**
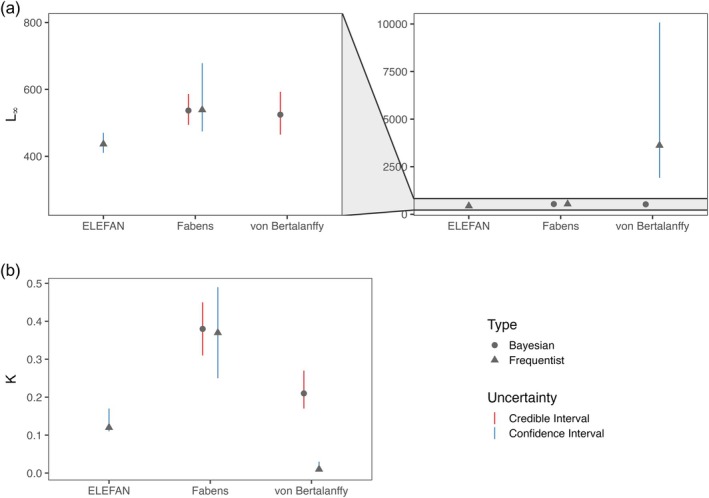
Parameters (a) *L*_∞_ and (b) *K* estimated by the growth models after von Bertalanffy and Fabens and estimated by the ELEFAN approach. The von Bertalanffy and Fabens growth models were fitted to the data with a Bayesian and a frequentist version. Corresponding to the statistical approach, the values show a credible (red) or confidence (blue) interval. For a better comparability of the results for the asymptotic length (*L*_∞_) the area between 250 and 800 mm was enlarged (a).

Parameters estimated from the traditional von Bertalanffy growth model differed strongly between the two statistical approaches (Figure 3). The frequentist model estimated an unreasonably high *L*_∞_ value of 3620.30 mm with CIs that ranged from 1914.94 to 10,078.97 mm and a *K* value of 0.01 year^−1^, smaller than the *K* values estimated by the other growth models. In contrast, the Bayesian von Bertalanffy growth model with strong priors estimated more realistic results with an *L*_∞_ value of 524.46 mm, close to the *L*_∞_ values estimated by the Fabens growth model. The credible interval of the Bayesian von Bertalanffy growth model ranged from 464.84 to 592.83 mm, whereas it estimated a *K* value of 0.21 year^−1^ with a credible interval ranging from 0.16 to 0.26 year^−1^.

For the length‐frequency data, the ELEFAN approach estimated an *L*_∞_ of 436.50 mm with a credible interval from 410.40 to 470.12 mm and a *K* value of 0.12 year^−1^. Compared to the other growth models, the ELEFAN approach resulted in the lowest *L*_∞_ value and the lowest *K*, excluding the value for *K* estimated by the frequentist von Bertalanffy, which is unreasonably low.

## 4 DISCUSSION

**FIGURE 4:**
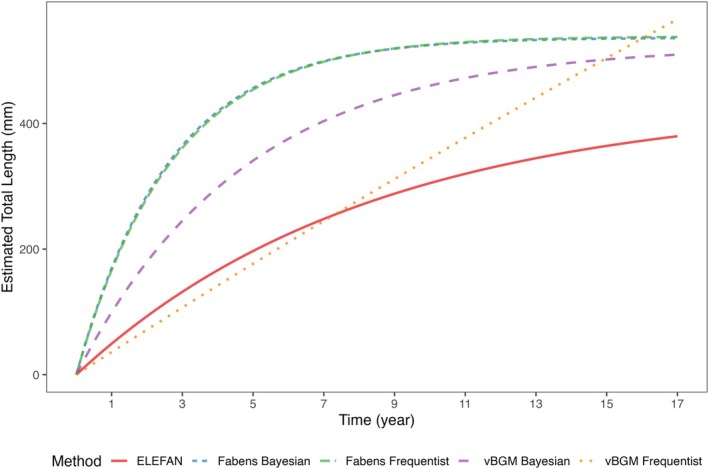
Growth curves based on the parameters estimated by the different growth models. For an easy comparison the *t*_0_ parameters of the von Bertalanffy growth model (vBGM) and the electronic length‐frequency analysis (ELEFAN) were set to zero.

Reliable estimates of growth build the fundament of most population/stock assessments. Density‐dependent and environmental factors such as temperature, food supply, and carrying capacity can lead to variations in the growth rate and asymptotic length of fish populations (Fogarty & Collie, 2020; Lorenzen, 2016; Lorenzen & Enberg, 2002). Therefore, it is important to estimate fish growth when working in regions lacking specific growth data. In this study, we provide a first reliable growth model for the European grayling in the alpine region of Germany. For this purpose, we used three different datasets and growth models: a von Bertalanffy growth model with age‐at‐length data (dataset 1) obtained from scales, Faben's growth model with length increment data (dataset 2) from mark and recapture events, and the ELEFAN approach with length‐frequency data (dataset 3). Except for the frequentist approach of the traditional von Bertalanffy growth model to the age‐at‐length data, resulting in a too high *L*_∞_ value and a very low *K* value, the different approaches gave reasonable results for all three datasets (see Figure 4).

However, comparing the results of the traditional vBGM and the one obtained by mark‐recapture data is not advisable as the asymptotic length estimated by both models does not have the exact same meaning (Francis, 1988). In the growth model, after Fabens, the asymptotic length will be the maximum observed length, but in the vBGM, it is the mean maximum length, and fish that are bigger than this value can be observed (Francis 1982). A simulation study by Haddon (2011) showed that the asymptotic length estimated by the growth model after Fabens tends to be usually higher than that estimated with the vBGM and age‐at‐length data. The same can be observed with our results, where the asymptotic length of both models differed by about 15 mm. Nevertheless, studies with mark‐recapture and age‐at‐length data could show that both models usually result in comparable results (Erhardt & Scarnecchia, 2013; Sheffer et al., 2022). The frequentist version of the vBGM resulting in an unreasonably high asymptotic length and a very low growth coefficient *K* could result from under‐aging of the scales. Scales are often considered unreliable, and under‐aging occurs frequently, especially with older fish, where annuli tend to be closer together and more challenging to identify (Quist & Isermann, 2017). Horká et al. (2010) found that in the grayling in southwest England, the ages 1+ and 2+ were reliably identified, but the accuracy of the aging process declined with increasing age. The Bayesian version of the vBGM with strong priors in contrast to the frequentist approach resulted in acceptable results for the parameters and underlined the importance of the Bayesian approach in situations where age‐at‐length data might be biased or limited (Doll & Jacquemin, 2018). The maximum age for grayling in this study was 7 years, with the most individuals between the ages 0 and 3. In general, the grayling is considered a short‐lived species, and maximum ages from the U.K. are reported to be 6–7 years from the chalk streams Llyn Tegid and River Dee (Woolland & Jones, 1975), which is in line with our findings from the River Inn where the oldest fish was also 7 years. The grayling inhabiting the chalk streams in the U.K. are reported to grow extremely fast, and the YOY have been reported with sizes between 100 and 150 mm (Woolland & Jones, 1975). In our study, the YOY were even found to have considerably larger maximum sizes ranging from 136 to 233 mm. Older specimens have been reported from former Yugoslavia at 13 years (Horká et al., 2010) and at 14 years in Germany (Muus & Dahlström, 1968), twice as old as the maximum age of this study. The growth coefficient *K* can give information on the productivity of a population; a *K* value >0.3 year^−1^ and between 0.3 and 0.16 year^−1^ show a high or medium productivity, respectively (Musick, 1999). Consequently, the growth model after Fabens would indicate a highly productive population with fast‐growing and short‐living individuals. In contrast, the Bayesian vBGM indicates a medium productivity, where individuals would reach higher ages.

Among the different growth models, the ELEFAN approach resulted in the smallest asymptotic length and growth coefficient (K). A problem that can arise when analysing length‐frequency data is that after maturation, the growth of fish typically slows down, making it hard to distinguish between age cohorts (Haddon, 2011). This was also confirmed by Schwamborn et al. (2019) who showed the tremendous importance of larger individuals when using the ELEFAN approach for estimating growth. Capturing more large individuals on every sampling occasion can have strong positive effects on the accuracy of the model. The length‐frequency data used for the analysis with the ELEFAN approach showed that most grayling were found in a size range from about 100.00 to 300.00 mm (see Figure 2c). Individuals over 400.00 mm were rare and not even present in some sampling events, albeit the River Inn is not free from recreational fishing. The fishing regulations in Germany mandate the harvesting of fish that exceed the minimum size limit, prohibiting catch and release practices (Arlinghaus, 2007). In heavily fished populations, larger and older fish are often rare, typically replaced by fast‐growing medium‐sized fish (Hilborn & Walters, 1992). The challenge in identifying older age cohorts and a lack of larger‐sized fish could explain why the asymptotic length and the growth coefficient are smaller than the ones predicted by the other models. Compared with other studies (Table 1), the asymptotic length of 430.48 mm seems coherent. However, the growth coefficient of 0.12 year^−1^, which indicates that the fish would need relatively long to reach the asymptotic length is in contrast with the results provided by the other studies.

Estimating the growth of fish is often considered the piece of cake part of most stock or population assessments. However, bias in the estimated parameters can have severe consequences on the outcome of these, for example, when estimating natural mortality (Pauly, 1980) or biomass (Francis, 2016). Our study on the growth of the grayling showed that different growth models working on different datasets can have different outcomes for the same population, especially when biased. Among the three datasets used for our study, the mark‐recapture data are likely to be the most unbiased and, therefore, give the most reliable growth model for the grayling in the alpine region of Germany. We could not confirm our initial hypothesis that the exclusive use of scale samples is sufficient to determine the correct age of grayling in our study, as it is even questionable if the younger ages are identified correctly by counts of annuli. As mentioned, the asymptotic length and, therefore, the growth coefficient *K* are higher when estimated by the growth model after Fabens. When using the estimated parameters in further analysis, it is, therefore, advisable to use a range of plausible values for the parameters (Fitzgerald et al., 2023). Using a Bayesian version of the mark‐recapture model, the uncertainty delivered by the credible intervals can give a range of reliable starting points for the following analysis.

## 5 CONCLUSION

Considering intensified efforts of fish conservation and habitat restoration, evidence‐based approaches and the use of valid indicators for the success or failure of measures are needed (e.g., Geist & Hawkins, 2016; Pander & Geist, 2013). As demonstrated in this case study on grayling from the River Inn, the use of growth characteristics may be a currently underestimated yet very useful indicator of target species assessment that complements other population health indicators such as population density and demography. Specifically, growth estimates can be used to determine age‐at‐maturation, habitat quality, and productivity, and consequently support fisheries management decisions such as the implementation of size limits. The observed high growth coefficient of grayling in the River Inn indicates a faster than expected life history, likely resulting in quick responses to conservation and restoration measures. The much slower growth of grayling in other areas of its distribution range suggests the need for a regionally differentiated management approach. At the same time, knowledge of growth characteristics from healthy populations provides a useful benchmark for comparison and early warning systems for other populations. Depending on the data source, the choice of only one single growth model can lead to over‐ or underestimation of growth compared to the approach chosen herein, where multiple data sources and analytical pathways are considered. As also shown in this study, decisions on the appropriate growth modeling approaches can benefit from an initial consideration of multiple data sources to help identify the most targeted and efficient approach.

## AUTHOR CONTRIBUTIONS

Conceptualization: Jan Droll, Christoffer Nagel, Juergen Geist; Methodology: Jan Droll, Christoffer Nagel, Juergen Geist; Formal analysis: Jan Droll; Investigation: Jan Droll, Sophie Ebert; Data curation: Jan Droll, Christoffer Nagel; Writing—original draft: Jan Droll; Writing—review and editing: Jan Droll, Christoffer Nagel, Joachim Pander, Sophie Ebert, Juergen Geist.

## FUNDING INFORMATION

This study received funding from Verbund Innkraftwerke GmbH in collaboration with the Bavarian State Ministry of the Environment and Consumer Protection.

## CONFLICT OF INTEREST STATEMENT

The authors declare that they have no competing interests.

## Data Availability

Data are available from the corresponding author upon reasonable request.
